# Direct Anandamide Activation of TRPV1 Produces Divergent Calcium and Current Responses

**DOI:** 10.3389/fnmol.2017.00200

**Published:** 2017-06-21

**Authors:** Axel J. Fenwick, Daniel K. Fowler, Shaw-Wen Wu, Forrest J. Shaffer, Jonathan E. M. Lindberg, Dallas C. Kinch, James H. Peters

**Affiliations:** Department of Integrative Physiology and Neuroscience, Washington State UniversityPullman, WA, United States

**Keywords:** permeability, vagus, calcium, glutamate, spontaneous, quantal, autonomic reflexes

## Abstract

In the brainstem nucleus of the solitary tract (NTS), primary vagal afferent neurons express the transient receptor potential vanilloid subfamily member 1 (TRPV1) at their central terminals where it contributes to quantal forms of glutamate release. The endogenous membrane lipid anandamide (AEA) is a putative TRPV1 agonist in the brain, yet the extent to which AEA activation of TRPV1 has a neurophysiological consequence is not well established. We investigated the ability of AEA to activate TRPV1 in vagal afferent neurons in comparison to capsaicin (CAP). Using ratiometric calcium imaging and whole-cell patch clamp recordings we confirmed that AEA excitatory activity requires TRPV1, binds competitively at the CAP binding site, and has low relative affinity. While AEA-induced increases in peak cytosolic calcium were similar to CAP, AEA-induced membrane currents were significantly smaller. Removal of bath calcium increased the AEA current with no change in peak CAP currents revealing a calcium sensitive difference in specific ligand activation of TRPV1. Both CAP- and AEA-activated TRPV1 currents maintained identical reversal potentials, arguing against a major difference in ion selectivity to resolve the AEA differences in signaling. In contrast with CAP, AEA did not alter spontaneous glutamate release at NTS synapses. We conclude: (1) AEA activation of TRPV1 is markedly different from CAP and produces different magnitudes of calcium influx from whole-cell current; and (2) exogenous AEA does not alter spontaneous glutamate release onto NTS neurons. As such, AEA may convey modulatory changes to calcium-dependent processes, but does not directly facilitate glutamate release.

## Introduction

Primary vagal afferent neurons relay satiety information to the brain and initiate coordinated autonomic and gastrointestinal reflex pathways (Saper, 2002; Berthoud, 2008). Most vagal afferents are unmyelinated or lightly myelinated C- and Aδ-fibers which extensively express the transient receptor potential vanilloid subfamily member 1 (TRPV1) throughout the neuron (Holzer, 1991). According to immunohistochemical and functional data, TRPV1 is expressed at the peripheral terminals, in the cell bodies of the nodose ganglion and centrally at the synaptic contacts in the brainstem nucleus of the solitary tract (NTS; Marsh et al., 1987; Doyle et al., 2002; Holzer, 2006; Czaja et al., 2008). TRPV1 activation at the central endings selectively controls quantal forms of glutamate release including action-potential independent spontaneous release and action-potential dependent asynchronous release (Wu et al., 2014). Capsaicin (CAP) activation of TRPV1 dramatically increases the frequency of spontaneous vesicle fusion and release, presumably through the direct influx of calcium ions (Andresen et al., 2012). However, TRPV1 is also permeable to sodium ions which contribute to membrane depolarization and recruitment of additional calcium permeant pathways. TRPV1-facilitated glutamate release targets a discrete pool of vesicles under some experimental conditions (Peters et al., 2010). However, sustained TRPV1 activation dramatically increases spontaneous release while depleting other vesicle release pathways; including action-potential driven synchronous release (Doyle et al., 2002). The extent to which TRPV1 fluxed calcium is required for TRPV1 to control glutamate release is unknown.

In addition to CAP, TRPV1 is activated by both exogenous and endogenous stimuli including high temperature, acidic pH, voltage and membrane lipids such as anandamide (AEA) and 2-arachidonoyl glycerol (2-AG; Clapham et al., 2001). Membrane lipids may serve as a predominant endogenous ligand at TRPV1 considering the relative stability of temperature and pH within the central nervous system. Peripherally, AEA is reported to sensitize and activate vagal afferents via a TRPV1 dependent mechanism (Kagaya et al., 2002; Lee et al., 2003; Lin et al., 2009). In the central nervous system, postsynaptic generation and release of AEA can act presynaptically via cannabinoid receptors to inhibit glutamate release (Kreitzer and Regehr, 2002) and may also activate TRPV1 in synapses where it is expressed. Presynaptic terminals may also generate local high concentrations of AEA (Di Marzo et al., 1994) which may be sufficient to activate TRPV1 locally and facilitate glutamate release. While CAP activation of TRPV1 produces large effects on quantal neurotransmission, the extent to which AEA activation of TRPV1 alters quantal forms of release from primary afferents to NTS neurons has not been characterized.

Here we compare the ability of AEA to activate TRPV1 to that of CAP and investigate the neurophysiological consequences on quantal forms of glutamate release. Using fluorescent calcium imaging and whole-cell patch-clamp recordings in dissociated nodose ganglion neurons, we conclude that AEA activation of TRPV1 produced large calcium responses but minimal whole-cell currents, whereas CAP activation produced robust calcium responses and large inward currents. Taking advantage of these differential effects, we tested the hypothesis that AEA induced calcium influx via TRPV1 directly controls glutamate release at the central synapses. However, we observed that AEA did not change spontaneous glutamate release in contrast to CAP activation of TRPV1. We speculate AEA may change calcium-dependent modulatory processes via TRPV1 but does not directly facilitate glutamate release.

## Materials and Methods

### Animals

Male Sprague Dawley rats (120–250 g, Simonsen Laboratories) and male TRPV1 knockout mice (TRPV−/−) (20–30 g) (B6.129X1-*Trpv1*^tm1Jul^/J, Jackson Laboratories) were used under procedures approved by the IACUC at Washington State University. Animals were housed under 12 h light/12 h dark conditions and fed standard pellet chow *ad libitum*. This study was carried out in accordance with the recommendations of the Guide for the Care and Use of Laboratory Animals, as drafted, updated and published by National Research Council.

### Molecular Biology

TRPV1−/− mice were genotyped with genomic DNA isolated from tail fragments using NaOH extraction. Primers and cycling parameters used to detect TRPV1 fragments from genomic DNA were obtained from the Jackson Laboratory website. For genotyping we used standard PCR with the following primers: common—TCC TCA TGC ACT TCA GGA AA; Wild type—CCT GCT CAA CAT GCT CAT TG; Mutant—TGG ATB TGG AAT GTG TGC GAG. The cycling parameters were: (1) 94°C × 3 min; (2) 94°C × 30 s; (3) 63°C × 1 min; (4) 72°C × 1 min; (5) Repeat steps 2–4 × 35 cycles; (6) 72°C × 2 min; (7) 4°C hold.

### Nodose Ganglion Isolations and Primary Neuronal Cultures

Nodose ganglia were isolated bilaterally from rats and mice under a deep plane of anesthesia (Ketamine, 25 mg/100 g; with Xylazine, 2.5 mg/100 g) using aseptic surgical conditions previously reported (Lancaster and Weinreich, 2001; Simasko et al., 2002). Following a midline incision in the neck, the musculature was retracted and blunt dissection techniques were used to dissociate the vagal trunk from the common carotid artery. High-magnification optics (10–100× dissecting scope; Leica Microsystems, Buffalo Grove, IL, USA) were utilized to visualize the nodose ganglia and facilitate complete removal. Once isolated, nodose ganglia were desheathed and digested in Ca^2+^/Mg^2+^ free Hank’s Balanced Salt Solution containing 1 mg/mL of both Dispase II (Hoffmann La Roche) and Collagenase Type 1A (Sigma Aldrich; 90 min at 37°C in 95% air/5% CO_2_). Neurons were dispersed by gentle trituration through silicanized pipettes, and then washed in Dulbecco’s Modified Eagle’s Medium (DMEM) supplemented with 10% fetal bovine serum (FBS) and 1% penicillin-streptomycin. Dispersed cells were plated onto poly-lysine coated coverslips and maintained in DMEM+10% FBS (37°C in 95% air/5% CO_2_). Measurements were made within 24 h of isolation.

### Molecular Cloning, COS-7 and HEK293 Cell Culture and Transfection

To generate TRPV1-GFP, the full-length rat TRPV1 coding region (NCBI accession number NM_031982.1) without stop codon was PCR amplified from nodose ganglion cDNA using the primers below and inserted into the HindIII and EcoRI sites of pEGFP-N1 (Clontech).

Primer sequences:

- 5^′^-AGCTAAGCTTCCACCATGGAACAACGGGCTAGC-3^′^
- 5^′^-CCATGAATTCCTTTCTCCCCTGGGACCAT-3^′^

Clonal COS-7 cells (ATCC® Manassas, VA, USA) were plated on glass coverslips at a density of 250,000 cells per well of a 6-well tissue culture plate. HEK293 cells (ATCC® Manassas, VA, USA) were plated on glass coverslips at a density of 50,000 cells per well of a 12-well plate. COS-7 and HEK293 cells were maintained in DMEM (Invitrogen), 10% FCS (Atlanta Biologicals), 25 units/ml penicillin and 25 μg/ml streptomycin (Sigma). Approximately 24 h after plating, media was replaced with DMEM, 10% FCS without pen/strep and cells were transiently transfected overnight. COS-7 cells were transfected with 2 μg of TRPV1-GFP and 2.4 μl lipofectamine 2000 (Invitrogen) per well. HEK293 cells were transfected with 1 μg of TRPV1-GFP and 1.2 μl lipofectamine 2000 per well. COS-7 cells were used for calcium imaging and HEK293 cells were used for electrophysiological recordings 1–2 days following transfection.

### Ratiometric Fluorescent Calcium Measurements

Intracellular calcium measurements were made with the fluorescent Ca^2+^ indicator Fura-2 AM (Molecular Probes, Eugene, OR, USA). Manipulations were made at room temperature in a physiological saline bath (in mM: 140 NaCl, 5 KCl, 2 CaCl_2_, 1 MgCl_2_, 6 glucose, 10 HEPES with pH adjusted to 7.4 with NaOH). High K^+^ bath (HiK) had 55 mM KCl with an equimolar reduction of NaCl to 90 mM. Neurons on coverslips were loaded with 1 μM Fura-2-AM for 1 h followed by a 15 min wash for de-esterification. COS-7 cells on coverslips were loaded with 1 μM Fura-2-AM for 20 min followed by a 10 min was for de-esterification. Coverslips were mounted into a closed chamber and constantly perfused with physiological bath. Fluorescence was collected using a Nikon Eclipse Ti inverted microscope (Nikon Instruments Inc., Melville, NY, USA) with 40× oil immersion objective, and an Andor Zyla sCMOS digital camera (Andor, South Windsor, CT, USA). For COS-7 cell experiments, TRPV1-GFP+ cells were identified by imaging using an EGFP filter cube (Nikon) prior to Fura-2 imaging. Cells containing Fura-2 were alternatively excited with 340 nm and 380 nm light and fluorescence monitored at 510 nm. Data points were collected with MetaFluor software at 6 s time points. Ratios of fluorescence intensity were converted to Ca^2+^ concentrations with a standard curve. Peak calcium responses were quantified, analyzed and compared statistically.

### Whole Cell Patch-Clamp Recordings

Whole cell recordings were performed on dissociated nodose ganglion neurons or transfected HEK293 cells using an inverted Olympus IX50 microscope and on NTS neurons contained in horizontal brainstem slices using an upright Nikon FN1 microscope. For HEK293 cell experiments, TRPV1-GFP+ cells were identified visually using 488 nm light illuminations prior to patching. Recording electrodes (2.8–3.8 MΩ) were filled with an intracellular solution containing (mM): 10 CsCl, 4 CsOH, 110 Cs-methanesulfonate, 11 EGTA, 1 CaCl_2_, 2 MgCl_2_, 10 HEPES, 2 MgATP, and 0.2 MgGTP. The intracellular solution was pH 7.4 and 296 mOsm. All cells were studied under voltage clamp conditions with an Axopatch 200A or MultiClamp 700A amplifier (Molecular Devices, Union City, CA, USA). Neurons were held at *V*_H_ = −60 mV using pipettes in whole cell patch configuration. Signals were filtered at 3 kHz and sampled at 30 kHz using p-Clamp software (version 10, Molecular Devices). Liquid junction potentials were not corrected. Extracellular solution (artificial cerebral spinal fluid, aCSF) was continuously perfused and specific drugs were either bath applied (slice) or locally applied using a fast-step perfusion system (Warner Instruments, Hamden, CT, USA).

### Horizontal Brainstem Slice Preparation

Brainstem slice experiments were performed on rats anesthetized with isoflurane as previously described (Doyle et al., 2004). The medulla was removed from just rostral to the cerebellum to the first cervical vertebrae and placed in ice-cold aCSF containing (mM): 125 NaCl, 3 KCl, 1.2 KH_2_PO_4_, 1.2 MgSO_4_, 25 NaHCO_3_, 10 dextrose, and 2 CaCl_2_, bubbled with 95% O_2_–5% CO_2_. Once chilled and firm, the tissue was trimmed to remove the cerebellum. A wedge was taken from the ventral surface causing the brainstem to sit slightly to the right (or left if desired) when horizontal and orienting the solitary tract (ST) afferent axons with the NTS in a common plane for cutting. The tissue block was then mounted horizontally to a pedestal with cyanoacrylate glue and submerged in cold aCSF on a vibrating microtome (Leica VT1200S). Approximately 300 μm was removed from the dorsal surface and then a single 250 μm thick horizontal slice was collected containing the ST together with the neuronal cell bodies of the medial NTS region. Slices were cut with a sapphire knife (Delaware Diamond Knives, Wilmington, DE, USA) and secured using a fine polyethylene mesh in a perfusion chamber with continuous perfusion of aCSF bubbled with 95% O_2_–5% CO_2_ at 32–33°C and 300 mOsm.

### Functional Identification of Second-Order NTS Neurons

To selectively activate ST afferent fibers, a concentric bipolar stimulating electrode (200 μm outer tip diameter; Frederick Haer Co., Bowdoinham, ME, USA) was placed on distal portions of the visible ST rostral to the recording region. Constant current shocks to the ST occurred every 6 s (shock duration 60 μs) using a Master-8 isolated stimulator (A.M.P.I., Jerusalem, Israel). Suprathreshold shocks caused long latency excitatory postsynaptic currents (EPSCs). These evoked EPSCs result from the action-potential driven synchronous release of glutamate vesicles activating postsynaptic α-amino-3-hydroxyl-5-methyl-4-isoxazole-propionate (AMPA) type glutamate receptors in these recording conditions. Latency is the time between the shock artifact and onset of the synchronous EPSC. Synaptic “jitter” is the standard deviation of ST-EPSC latencies for 30 trials. Jitters of <200 μs identify monosynaptic afferent inputs onto the NTS neuron (Doyle and Andresen, 2001). The TRPV1 selective agonist CAP was applied to identify ST afferents as possessing TRPV1. Spontaneous EPSCs (sEPSCs) were measured in the absence of tetrodotoxin (TTX) whereas mini EPSCs (mEPSCs) were measured in the presence of TTX (1 μM).

### Statistical Analyses

#### Calcium Imaging Experiments

For each experiment, data were collected from three to four nodose ganglion cell cultures or plating of the clonal cells (COS-7). When possible, protocols were designed to be within subject and analyzed using repeated measures ANOVA followed by *post hoc* comparisons against control. Otherwise unpaired *T*-tests were used to compare between groups. Parameters of dose-response relationships (EC_50_, slope, maximum) were determined by sigmoid fit of the data. For antagonist studies (ruthenium red (RR), etc.) all neurons received each treatment and were compared using within subject *t*-tests. Data are expressed as the average ± SEM. Statistical analysis was performed using SigmaStat software (Systat Software Inc., San Jose, CA, USA).

#### Electrophysiological Experiments

Whole cell currents taken from isolated nodose ganglion cells or transfected HEK 293 cells were normalized to the membrane capacitance to control for variability in cell size. Statistical tests for experiments run on isolated neurons are as described above. For brainstem slice recordings the digitized waveforms of synaptic events were analyzed using an event detection and analysis program (MiniAnalysis, Synaptosoft, Decatur, GA, USA) for all quantal synaptic currents and Clampfit 10 (Molecular Devices) for all ST-stimulated currents. All events >10 pA were counted for frequency values. Fitting of quantal EPSC amplitudes and decay kinetics (90–10%) was performed using a fitting protocol (MiniAnalysis) on >100 discrete events. For statistical comparisons *T*-tests, Mann Whitney Rank Sum test, and linear regression analysis were used when appropriate (Systat Software Inc., San Jose, CA, USA). For group comparisons, *p* < 0.05 was considered statistically significant.

### Chemicals and Drugs

The chemicals and drugs used for this series of studies were purchased from retail distributors. Specifically CAP, AEA, SB366791, RR, TTX and Fura-2 AM were sourced from Tocris. The general salts used for making bath solutions were purchased from Sigma-Aldrich.

## Results

**Figure 1:**
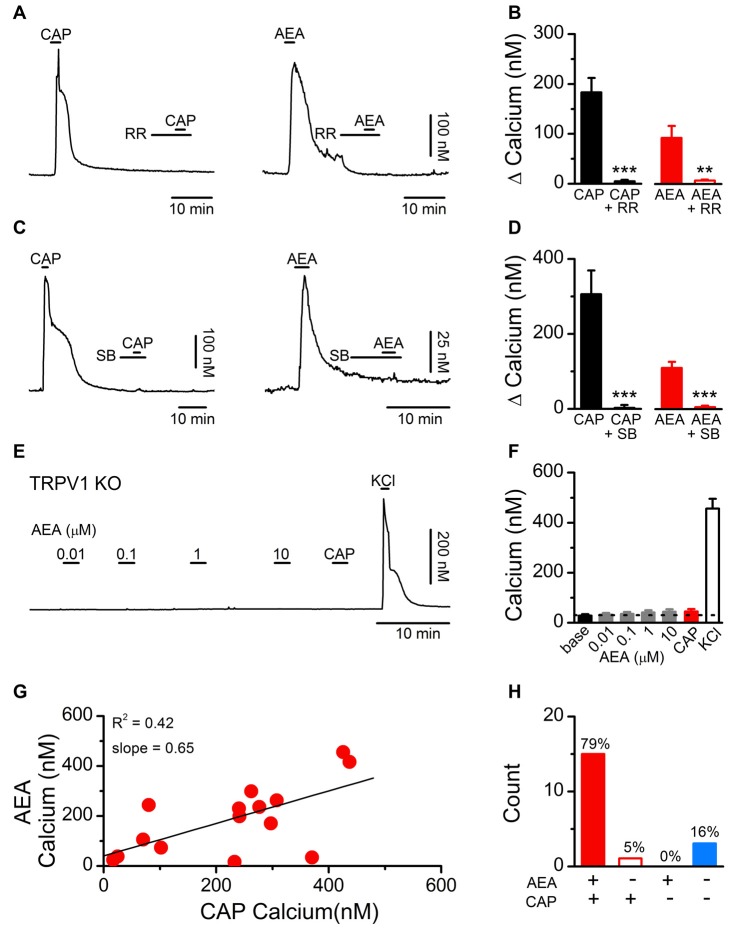
Establishing the dependence of anandamide (AEA) induced activation on transient receptor potential vanilloid subfamily member 1 (TRPV1) channels. **(A)** Representative calcium traces showing capsaicin (CAP; 100 nM) and AEA (10 μM) responses blocked by pretreatment with ruthenium red (RR, 1 μM). **(B)** Average CAP and AEA calcium responses before and following pretreatment with RR (CAP: *n* = 11, *P* < 0.001, *t*-test; AEA: *n* = 12, *P* = 0.005, *t*-test). **(C)** Similarly, the competitive TRPV1 antagonist SB366791 (SB, 10 μM) blocked both CAP and AEA induced increases in cytosolic calcium suggesting the effects of AEA are mediated via, or interact with, the intracellular CAP binding site. **(D)** Average CAP and AEA calcium responses before and following pretreatment with SB (CAP: *n* = 5, *P* = 0.007, *t*-test; AEA: *n* = 11, *P* < 0.0001, *t*-test). **(E,F)** No concentration of AEA tested produced an increase in cytosolic calcium concentrations in vagal afferent neurons taken from TRPV1 KO mice (*n* = 15). Recorded neurons were also tested with CAP (100 nM) to confirm the complete loss of TRPV1 and increased K^+^ to produce membrane depolarization and confirm cell viability. **(G)** The CAP responses were proportional to the AEA response (*n* = 15, slope = 0.65 ± 0.21, *R*^2^ = 0.42). **(H)** Across neurons AEA and CAP responses segregated (*n* = 22, *P* < 0.001, Fisher’s Exact). **P < 0.01, ***P < 0.001.

We initially determined if the stimulatory effects of AEA on dissociated vagal afferent neurons were mediated by TRPV1 as previously reported (Zygmunt et al., 1999; Smart et al., 2000) or via another cellular mechanism (van der Stelt and Di Marzo, 2005). For the AEA experiments the CB1 receptor antagonist AM251 was present in all the baths. Using fluorescent calcium imaging we observed that both CAP- and AEA-induced increases in cytosolic calcium were eliminated following treatment with the pore blocker RR (1 μM; Figures 1A,B) and by the competitive antagonist SB366791 (SB, 10 μM; Figures 1C,D) which is reported to occupy the intracellular orthosteric CAP binding site on TRPV1 (Gunthorpe et al., 2004). Time matched control experiments with repeated AEA showed a statistically significant attenuation of the second response but not the complete elimination seen with RR or SB366791 (1st AEA: 113 ± 26 vs. 2nd AEA: 43 ± 13, *N* = 12, *P* = 0.002, paired *T*-test). In vagal afferent neurons taken from TRPV1 KO mice, AEA failed to produce a calcium increase at any concentration tested (Figures 1E,F). Across vagal afferents taken from rats, responses to AEA were proportional to subsequent CAP responses and only occurred in CAP responsive neurons (Figures 1G,H). Taken together these data strongly suggest, and are consistent with previous findings, that in primary vagal afferent neurons the stimulatory actions of AEA require TRPV1 and occur via direct binding at sites interdependent with the CAP binding site (Ross, 2003).

**Figure 2:**
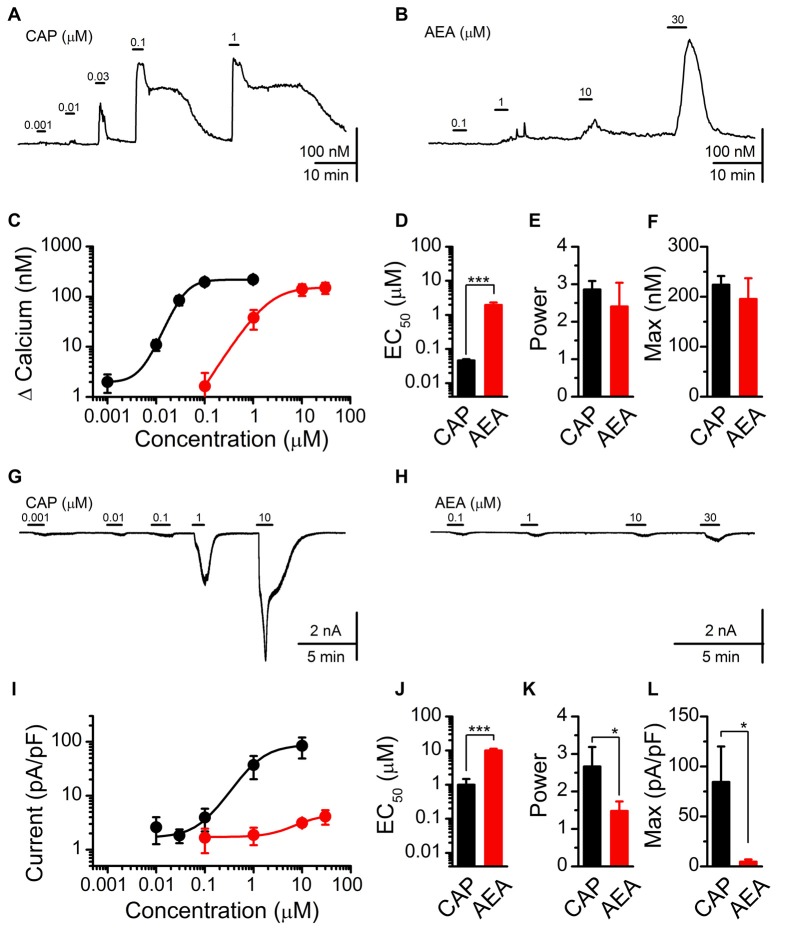
CAP and AEA activation of cultured vagal afferent neurons produces similar calcium increases but different current responses.** (A,B)** Representative traces showing transient calcium influxes from concentration dependent activation with CAP (1 nM–1 μM) and AEA (100 nM–30 μM). **(C)** Average concentration response curves for CAP (*n* = 23) and AEA (*n* = 10) as characterized with calcium imaging. **(D–F)** AEA has a significantly higher EC_50_ than CAP (*P* < 0.001, *t*-test), but similar concentration response power (*P* = 0.41, *t*-test) and peak increase in cytosolic calcium (*P* = 0.45, *t*-test). **(G,H)** Concentration response relationships for CAP and AEA using whole-cell patch clamp electrophysiology. CAP produced large inward currents at high concentrations while AEA-evoked inward currents remained relatively small across concentrations. **(I)** Average inward current by concentration for CAP (*n* = 5) and AEA (*n* = 7) as measured at *V*_m_ = −60 mV in the voltage-clamp configuration. **(J–L)** AEA shows a statistically greater EC_50_ from CAP (*P* < 0.001, *t*-test), a decrease in slope (*P* = 0.05, *t*-test), and a much smaller maximal inward current (*P* = 0.02, *t*-test). *P < 0.05, ***P < 0.001.

Using fluorescent calcium imaging and whole-cell patch clamp electrophysiology, we next characterized and compared the stimulatory actions of AEA and CAP (Figure 2). Both CAP and AEA produced concentration-dependent increases in cytosolic calcium (Figures 2A,B). CAP activation produced a long lasting elevation in cytosolic calcium compared to AEA, likely due to sequestration into organelles and subsequent equilibration over time. For quantification, we measured the peak calcium influx following ligand exposure. The averaged response profiles showed similar maximum efficacy between CAP and AEA to increase peak calcium but dramatically different EC_50_ concentrations (Figure 2C). Fitting of the data confirmed a statistically larger EC_50_ value for AEA over CAP (Figure 2D), but no systematic differences in the power (Figure 2E) nor maximum cytosolic calcium were observed (Figure 2F). Using voltage-clamp configuration (*V*_m_ = −60 mV) CAP exposure produced concentration-dependent increases in inward current that became very large at the highest concentrations (Figure 2G), while AEA produced only very modest inward currents even at maximal concentrations (Figure 2H). The EC_50_ for CAP and AEA activation was notably different between calcium measurements and current recordings (CAP EC_50_ for Calcium: 0.046 ± 0.005 μM vs. for Current: 0.99 ± 0.47 μM, *P* < 0.001, *T*-test) (AEA EC_50_ for Calcium: 1.95 ± 0.36 μM vs. for Current: 9.80 ± 1.07 μM, *P* < 0.001, *T*-test) revealing differences in the detection of only calcium vs. whole cell currents.

**Figure 3:**
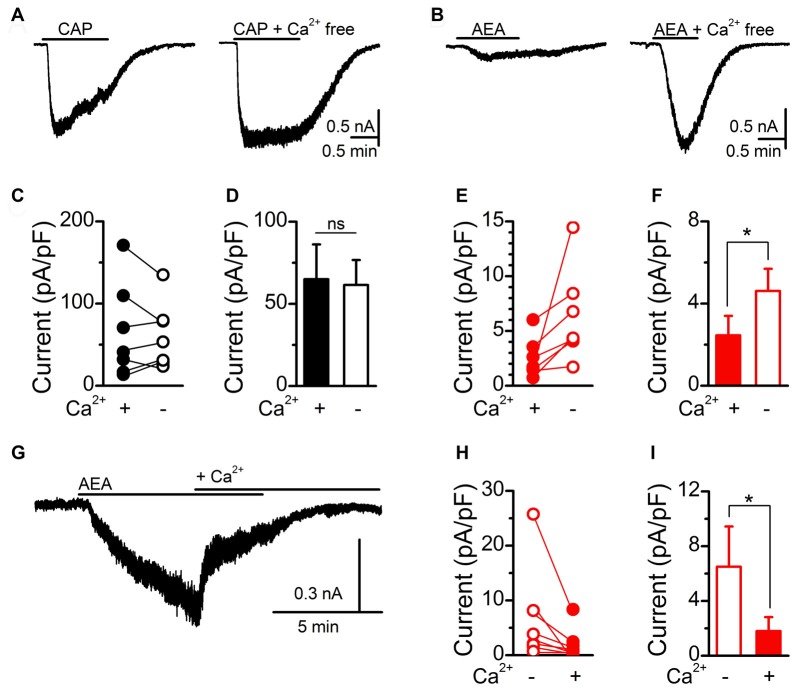
AEA activation of TRPV1 is sensitive to extracellular calcium.** (A,B)** Representative current traces showing saturating CAP (10 μM) and AEA (30 μM) induced currents in the presence (right) and absence (left) of bath calcium (2 mM). Removal of bath calcium did not change the maximum CAP (*n* = 8, *P* = 0.31, *t*-test) current but increased the AEA (*n* = 6, *P* = 0.049, *t*-test) induced current. Plots of individual and average CAP **(C,D)** and AEA** (E,F)** responses before and following removal of bath calcium. **(G)** The addition of calcium into the bath during an AEA exposure rapidly diminished the evoked inward current. **(H,I)** Plot of individual and average AEA responses in calcium free and calcium containing bath conditions. The addition of bath calcium rapidly reduced the AEA induced current (*n* = 8, *P* = 0.049, *t*-test). *P < 0.05.

To probe the discrepancy in AEA signaling between calcium imaging and current measurements, we next performed calcium exclusion experiments with whole-cell patch clamp recordings (Figure 3). Saturating CAP (10 μM) activation of TRPV1 produced large inward currents that rapidly desensitized in standard bath containing calcium. While removal of bath calcium did not change the average amplitude of CAP induced currents, it did eliminate the calcium dependent desensitization (Figures 3A,C,D; Cholewinski et al., 1993; Koplas et al., 1997). Saturating AEA (30 μM) activation of TRPV1 produced only small inward currents in standard bath that were significantly larger following removal of bath calcium (Figures 3B,E,F). To confirm the interaction of calcium with the induced current we applied AEA in a calcium free bath first and then switched to a calcium containing bath while AEA was still present (Figure 3G). Across AEA responses the addition of extracellular bath calcium significantly reduced the initial inward current (Figures 3H,I). These findings demonstrate that in the presence of extracellular calcium maximal CAP activation of TRPV1 appears unchanged while AEA activation is greatly reduced, suggesting differential calcium sensitivity between CAP or AEA activated TRPV1.

**Figure 4:**
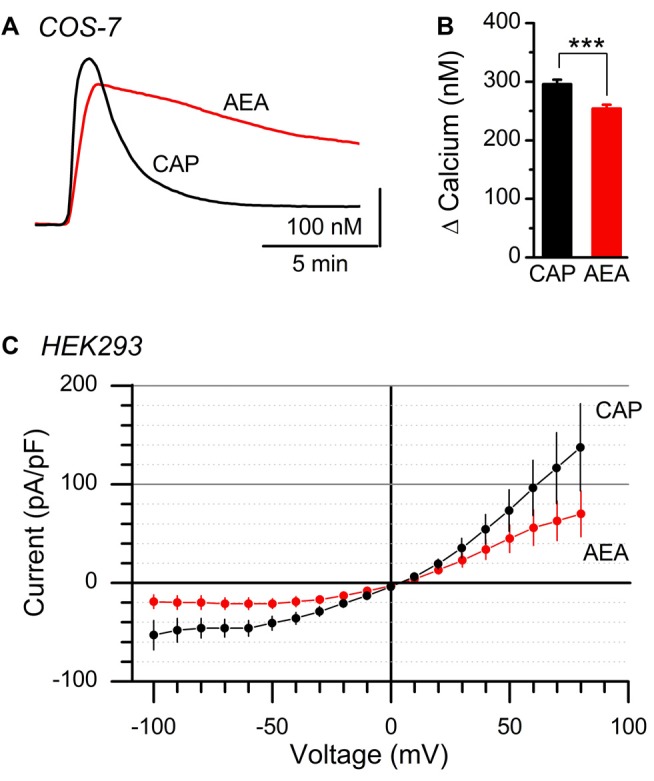
Comparison of AEA and CAP activation of isolated TRPV1. **(A)** Average calcium traces showing AEA and CAP activation of TRPV1 containing COS-7 cells. **(B)** Plot of average peak amplitudes across AEA (*n* = 453 cells) and CAP (*n* = 492 cells). AEA peak levels were statistically lower than CAP (*P* < 0.001, *t*-test). **(C)** Average current-voltage plots from AEA (*n* = 9 cells, red) and CAP (*n* = 9, black) induced activation of TRPV1 expressed in clonal HEK293 cells. Currents produced by both ligands reversed at 5 mV holding potential. ***P < 0.001.

Calcium can impact ion channel function via low pore permeability, diminished channel conductance, or via charge shielding/alteration of voltage sensors (Hille, 2001; Samways and Egan, 2011). Native vagal afferent neurons contain numerous ion channels in addition to TRPV1, making accurate determination of the current reversal potential challenging. We utilized overexpression of recombinant rat TRPV1 C-terminally conjugated to GFP (TRPV1-GFP) in heterologous cell lines lacking native TRPV1 expression to assay the effects of CAP and AEA at TRPV1 in isolation. Similar to native vagal afferent neurons, we found both CAP and AEA produced large calcium responses in COS-7 cells transfected with TRPV1-GFP (Figure 4A). Compared to CAP, AEA responses showed prolonged decay kinetics and slightly smaller (~14% smaller), but statistically different, peak response (Figure 4B). Current recordings in TRPV1-GFP transfected HEK293 cells with physiological bath confirmed that AEA produced smaller current changes (~52% smaller at *V*_m_ = −60 mV) than CAP, although proportionally larger than in native neurons. Both AEA and CAP currents reversed at identical potentials (Figure 4C), suggesting ion selectivity was not different between the ligands. Thus the ligand specific discrepancy between calcium flux and total current is not due to an anomalous mole fraction phenomenon, but rather involves ligand specific changes in gating or permeation as suggested previously (Samways et al., 2008).

**Figure 5:**
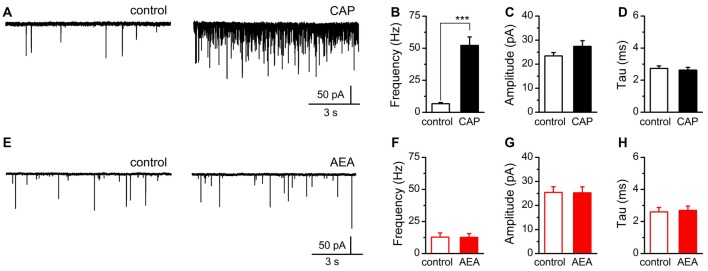
AEA fails to alter quantal glutamate release from central afferent terminals. **(A,E)** Representative current traces from a second-order nucleus of the solitary tract (NTS) neurons showing spontaneous glutamatergic excitatory postsynaptic currents (EPSCs) under control (left) and following CAP (right top, 1 μM) or AEA (right bottom, 30 μM) exposure. **(B–D)** Plots of average CAP responses showing significant increase in the rate of spontaneous glutamate release (*n* = 13, *P* < 0.001, *t*-test), but no significant change in amplitude (*n* = 13, *P* = 0.19, paired *t*-test), nor decay-time constant (*n* = 13, *P* = 0.65, paired *t*-test). **(F–H)** Plots of average AEA responses showing no significant change in spontaneous frequency (*n* = 16, *P* = 0.76, paired *t*-test), event amplitude (*n* = 16, *P* = 0.88, paired *t*-test), nor decay-time constant (*n* = 16, *P* = 0.33, paired *t*-test). ***P < 0.001.

Recognizing these differences in agonist activation of TRPV1, we sought to determine if AEA, independent of CB1, was sufficient to mobilize quantal glutamate vesicle fusion and release at central vagal afferent synapses. For this experiment we recorded spontaneous and miniature (with TTX) glutamate release onto identified second-order NTS neurons receiving direct innervation from primary afferents (Figure 5; Doyle and Andresen, 2001). Consistent with previous reports, we found a majority of vagal afferents were sensitive to CAP (1 μM) and produced a dramatic increase in spontaneous glutamate release following bath application with no significant changes in event amplitude or decay kinetics (Figures 5A–D; Andresen et al., 2012). In contrast, exposure to high concentrations of AEA (30 μM) failed to produce a significant change in any sEPSC parameter (Figures 5E–H).

## Discussion

In this study we investigated the ability of the endogenous cannabinoid AEA to directly bind to and gate native TRPV1 ion channels in primary vagal afferent neurons. In dissociated nodose ganglion neurons we found that AEA produced robust calcium influx but very little inward current, in contrast with CAP activation, which resulted in large calcium and current responses. While both CAP and AEA directly bound and activated TRPV1, the presence of extracellular calcium was only a key determinate of AEA signaling, but not CAP, suggesting specific differences in the TRPV1 signaling following ligand application. High concentration (saturating) CAP exposure was not sensitive to extracellular calcium while AEA activation of TRPV1 was inhibited by extracellular calcium. Isolation of the CAP and AEA activation of TRPV1 revealed identical current reversal potentials, suggesting this calcium influx vs. total current discrepancy was not due to differences in channel cation selectivity. Utilizing this difference, we determined that AEA signaling was insufficient to mobilize glutamate vesicles at the central terminals. Collectively, these data extend our understanding of AEA activation of TRPV1 and suggest its binding and gating preferentially favors calcium influx, an effect that is more dramatic in native vagal afferent neurons compared to clonal expression of TRPV1. This mode of activation provides evidence for ligand-dependent ion tuning, and, while AEA was unable to impact quantal glutamate release at the central terminals, it may still provide an important calcium influx pathway key to modulatory changes.

It is well established that AEA can serve as a direct ligand and indirect potentiator of TRPV1 signaling under experimental conditions (De Petrocellis and Di Marzo, 2005). However, despite a large body of literature the extent to which AEA may act at TRPV1 under endogenous conditions largely remain open questions (De Petrocellis and Di Marzo, 2009). Our current finding that AEA promotes calcium influx, with little change in total current or neurotransmission, may underlie the difficulty detecting endogenous AEA neurophysiological actions. While several reports have observed direct changes in glutamate release with application of exogenous AEA, the magnitude of the responses were small compared to CAP activation and required blockade of CB1 signaling in order to be detected (Palazzo et al., 2008; Bhaskaran and Smith, 2010; Boychuk et al., 2013). Sustained genetic inactivation of the degradative enzyme fatty acid amide hydrolase (FAAH) seems to provide some advantage in detecting ongoing AEA activity at TRPV1 (Musella et al., 2009); however, the generally minimal effects of AEA at TRPV1 are somewhat surprising given the characterization of AEA as a full agonist at TRPV1 in some instances. The characterization of AEA pharmacological activity is dependent on TRPV1 receptor abundance with full AEA agonism occurring in conditions when large amounts of excess TRPV1 are present (van der Stelt and Di Marzo, 2004). Additionally, it appears to matter which ion conductance is being monitored as to the AEA efficacy. We found monitoring calcium alone supports AEA as a full, albeit low affinity, agonist at native TRPV1. However, monitoring total current (including the passage of both sodium and calcium) indicates AEA is both a low affinity and low efficacy agonist at TRPV1 in native neurons and at negative holding potentials. As such, depending on the relative TRPV1 abundance or the ionic species of importance the effect of AEA may range from large to negligible.

Previous reports have documented dynamic, agonist induced, changes in ion channel pore selectivity for calcium and other large ions over sodium (Chung et al., 2008; Chen et al., 2009; Rokic and Stojilkovic, 2013; Munns et al., 2015); although the malleability of ion permeation of the pore is not without alternative interpretations (Bean, 2015; Li et al., 2015). One major critique of these experiments is the likelihood of significant ion accumulation intracellularly that may be sufficient to shift the relevant equilibrium potentials of permeant ions (Li et al., 2015; Samways et al., 2016). Here we report in native TRPV1 expressing neurons differential sensitivity to extracellular calcium between CAP and AEA activation of TRPV1. Given the relatively low abundance of native TRPV1 (compared to cell overexpression systems) and small AEA-induced currents the differential calcium influx appears to be dependent on the ability of the ligand to gate the channel. During vigorous activation, such as with high CAP concentrations, the impact of calcium is minimal or not present; whereas weaker activation, either by high AEA concentrations or lower CAP concentrations, produces a strongly calcium sensitive current. One possibility is that the removal of extracellular calcium may shift the voltage sensitivity of TRPV1, thus making the channel more excitable to low CAP and AEA exposure even at hyperpolarized potentials (Frankenhaeuser and Hodgkin, 1957; Nilius et al., 2007). Alternatively, the significantly lower EC_50_ calculations from calcium imaging data vs. the whole-cell currents suggest the effect of calcium is on channel conductance, with low efficacy activation preferentially fluxing calcium. This scenario suggests that the efficacy of the ligand or stimulus activating TRPV1 can influence not only the magnitude but predominant permeant ion, providing an elegant point of control over TRPV1 signaling to influence neurophysiological parameters. However, this interpretation is incomplete given that we found TRPV1 currents reverse at identical holding potentials with both CAP and AEA activation. Differences in channel gating predict divergence in ligand/receptor interactions of CAP and AEA that go beyond simple differences in affinity or ion selectivity. One possibility is the presence of additional allosteric binding for CAP that is not accessible to AEA and distinct from the shared orthosteric pocket previously described (Yang et al., 2015). TRPV1 has multiple points of activation and noted allosteric interactions between modalities of activation (Benham et al., 2002) and extracellular calcium concentrations (Samways and Egan, 2011). Systematic interrogation of the intracellular CAP binding site suggests nuanced differences in ligand occupancy when compared to resiniferatoxin that may also explain the discrepancy with AEA activation (Elokely et al., 2016).

Of particular interest to us is the ability of TRPV1 signaling to preferentially increase quantal forms of synaptic vesicle release, including spontaneous and asynchronous release pathways. It has been posited that TRPV1 calcium influx is specifically targeted to interact with a dedicated “spontaneous pool” of vesicles (Andresen et al., 2012) consistent with, and extending, our understanding of the intricate controls of vesicle release from the presynaptic terminal (Kavalali, 2015; Schneggenburger and Rosenmund, 2015). In this case, however, why then was the AEA-induced calcium influx incapable of increasing vesicle release? One possibility is that the AEA calcium influx was simply not of sufficient magnitude, although a dedicated TRPV1-to-vesicle targeting model would predict even some AEA-induced calcium should produce a graded change in the frequency of spontaneous release; an effect we failed to see. Alternatively, CAP induced increases in spontaneous release may require the additional sodium influx and subsequent depolarization to fully manifest its effects on spontaneous vesicle fusion and release. This would suggest the recruitment of additional voltage sensitive calcium channels. Although cadmium blockade does not eliminate temperature and CAP driven release (Jin et al., 2004; Shoudai et al., 2010); T-type calcium channels are less sensitive to extracellular cadmium blockade (Hille, 2001), are present in primary vagal afferent neurons (Mendelowitz and Kunze, 1992), and may resolve the CAP, but not AEA, increase in spontaneous vesicle release. In line with this possibility, the relatively weak AEA agonism of TRPV1 may be counteracted by its reported ability to inhibit voltage activated calcium channels and potassium channels (Chemin et al., 2001; Ross et al., 2009). Nevertheless, the net observed effect was that saturating concentrations of AEA produced no change in the frequency of spontaneous glutamate release from primary afferent terminals containing TRPV1, suggesting alternative signaling functions between CAP and AEA.

## Author Contributions

AJF and FJS made electrophysiological recordings, analyzed data and contributed to the writing/revision of the manuscript. S-WW collected calcium imaging data, analyzed recordings, contributed to the writing/revision of the manuscript. JEML and DCK collected electrophysiological and calcium imaging recordings, analyzed the data and contributed to the drafting and editing of the manuscript. DKF produced the TRPV1-GFP heterologous cell line, performed calcium imaging, electrophysiology and contributed to the writing and editing of the manuscript. JHP contributed to data collection (electrophysiology and calcium imaging), analyzed data and writing the manuscript. All authors contributed to the design and completion of the experiments.

## Conflict of Interest Statement

The authors declare that the research was conducted in the absence of any commercial or financial relationships that could be construed as a potential conflict of interest.
